# Case Report: A Chinese Family of Type A Insulin Resistance Syndrome With Diabetes Mellitus, With a Novel Heterozygous Missense Mutation of the Insulin Receptor Gene

**DOI:** 10.3389/fendo.2022.895424

**Published:** 2022-05-12

**Authors:** Wei You, Jianming Yang, Lu Wang, Yanqun Liu, Wen Wang, Li Zhu, Wei Wang, Jun Yang, Fangyuan Chen

**Affiliations:** Department of Endocrinology, The People’s Hospital of China Three Gorges University and The First People’s Hospital of Yichang, Hubei, China

**Keywords:** type A insulin resistance syndrome, insulin receptor gene, heterozygous missense mutation, diabetes, Chinese family

## Abstract

Type A Insulin resistance syndrome (TAIRS) is an autosomal dominant or recessive genetic disorder caused by insulin dysfunction resulting from insulin receptor (INSR) gene mutation. The main features of TAIRS include hyperinsulinemia, abnormal glucose metabolism, and changes in acanthosis nigricans. We identified, in China, a TAIRS family with a novel heterozygous missense gene mutation type. One patient from the Chinese Han family exhibited signs and symptoms of TAIRS and was presented for evaluation. Whole-exome sequencing revealed a heterozygous mutation. Both the patient proband and his father were identified with insulin receptor exon 19c.3472C>T(p.Arg1158Trp), which resulted in a missense mutation that led to replace by a base in the amino acid codon. We found that the patient proband and his father exhibited high insulin and C-peptide release after glucose stimulation by insulin and C-peptide release tests. At the same time, we also ruled out the possibility of islet βcell tumor through relevant examinations. These findings indicate that the INSR gene mutation may cause pancreatic β cell functional impairment and contribute to the development of diabetes.

## Introduction

TAIRS is an autosomal dominant or recessive genetic disorder caused by islet dysfunction due to mutations in the INSR, which mainly manifests as severe insulin resistance, hyperandrogenaemia, and acanthosis nigricans, mostly occurring in younger patients. Some patients enter regular states of hypoglycemia requiring differentiation from insulinoma. In this paper we describe, in detail, one Chinese case with the point mutation of gene c.3472C>T (p. Arg1158Trp). Here, we also describe a therapeutic approach that was employed for a patient with type 2 diabetes who had heterozygous missense mutations in the INSR. According to the diagnosis and treatment of TAIRS and the pathogenesis of INSR gene mutation, the clinician can pay attention to insulin resistance, timely diagnosis and treatment, and improve the prognosis.

## Case Description

**Figure 1:**
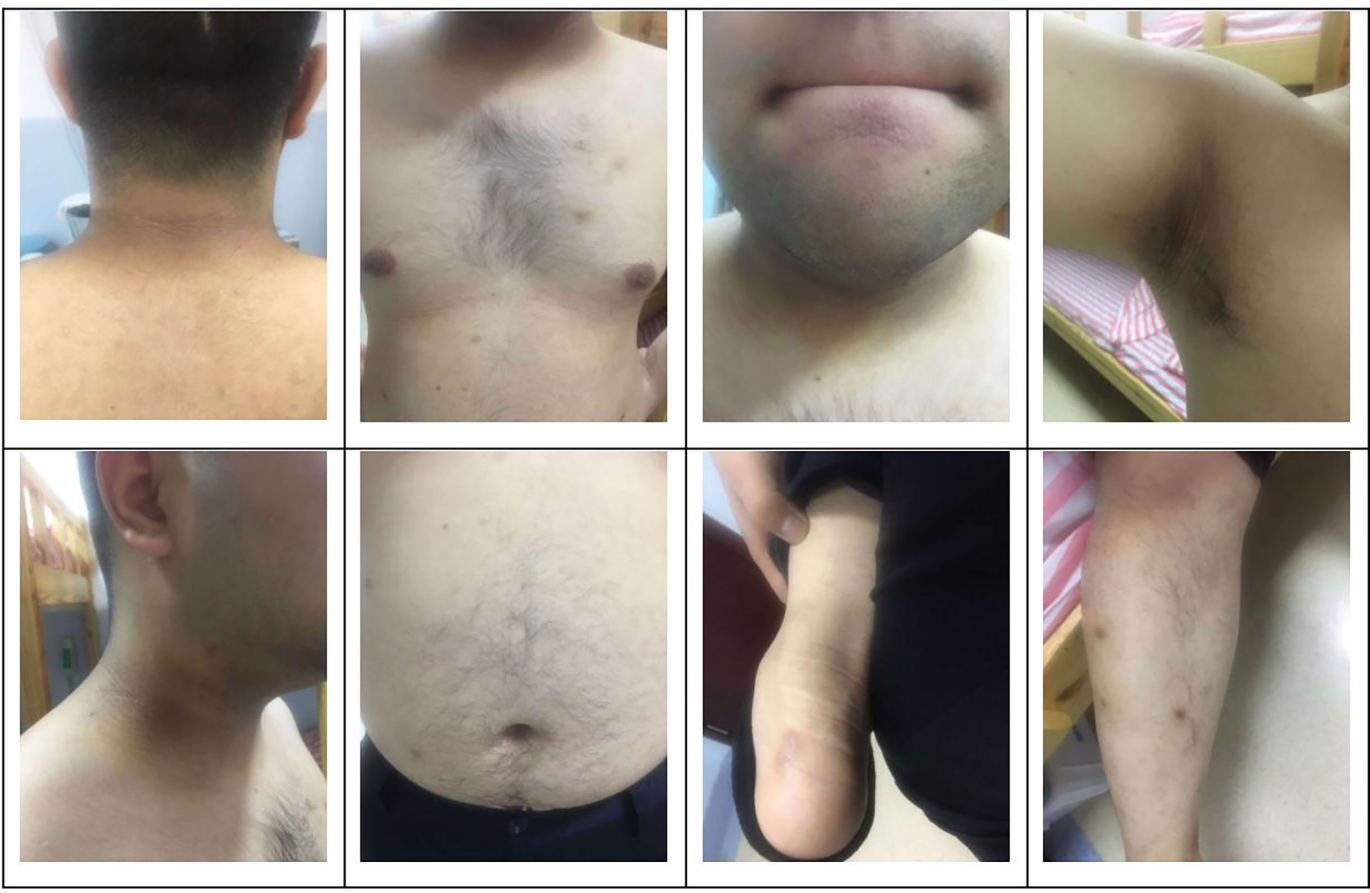
Clinical characteristics of the patient: obvious pigmentation in the skin around the neck, underarm pit and inguinal areas and rough hyperplasia with increasing hair in the chest and abdomen.

**Figure 2:**
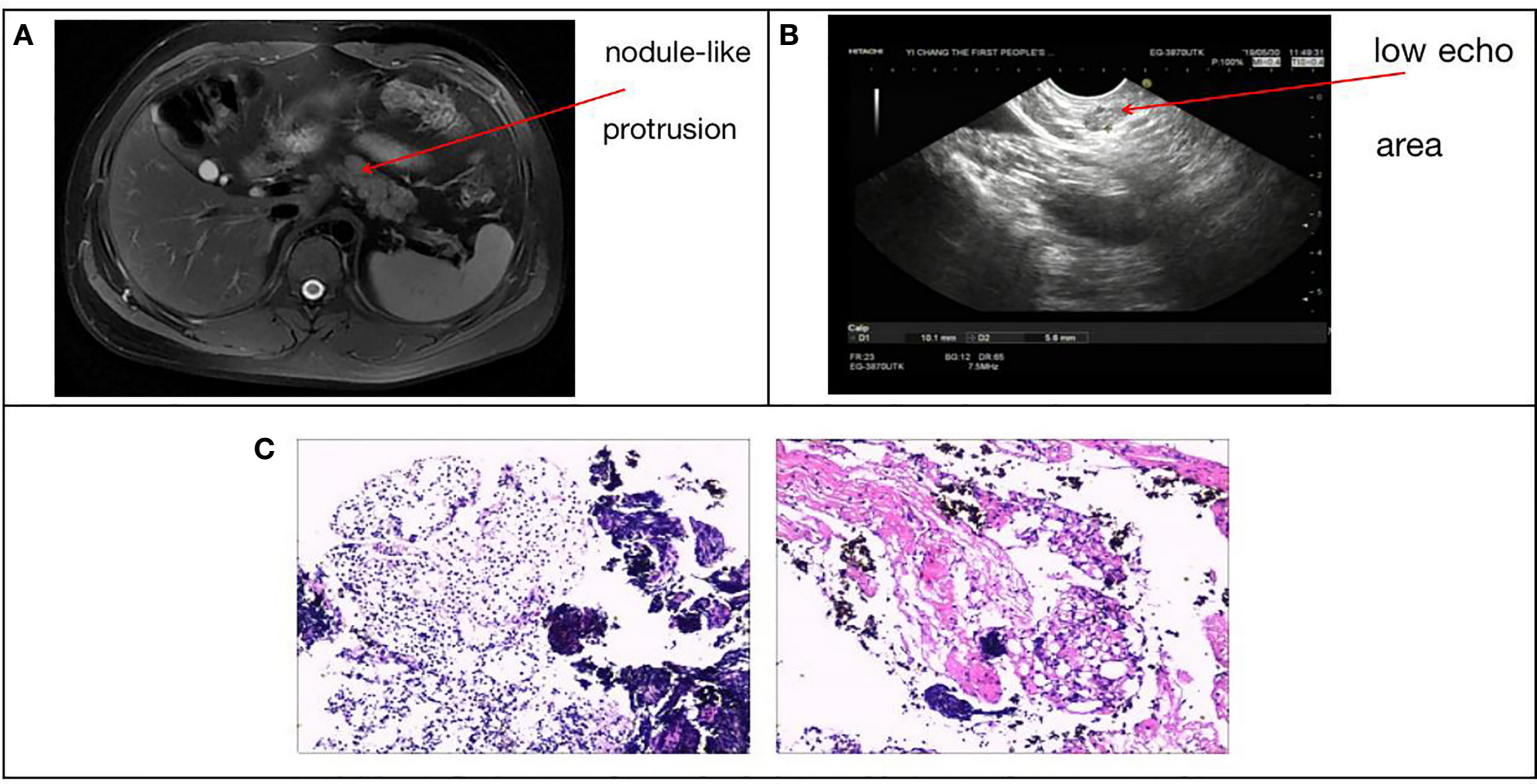
**(A)** Pancreatic MRI + enhancement: pancreatic neck diameter 6 mm nodule-like protrusion. **(B)** Patient ultrasound endoscopy: a low echo area in the pancreatic neck with a sectional size of approximately 1.0*0.6 cm. **(C)** EUS-FNA, intraoperative pancreatic tissue disease showed pancreatic puncture tissue is mostly cellulose-like exudate, large lymphocytes and little eosinophils diffuse or focal infiltration, local extrusion deformation, structure under clear, no pancreatic tissue structure and other were seen.

The subject of this case report is a 22 year old male patient who was admitted to the hospital due to repeated palpitation, sweating, hunger for 10 years, and weight gain for 2 months. The patient had a history of acanthosis nigricans. His grandfather was a diabetic and his uncle was a patient with adrenal cortical adenoma. The body size was obese. Physical examination showed obvious pigmentation in the skin around the neck, underarm pit, and inguinal areas and rough hyperplasia with increasing hair in the chest and abdomen (**Figure 1**) and other data were normal. Laboratory tests showed that the diabetic autoantibodies: anti-insulin antibody (IAA), anti-islet cell antibody (ICA), and anti-glutamate decarboxylase antibody (GAD) were all negative; insulin growth factor-1 (IGF-1), insulin growth factor-binding protein-3 (IGFBP3) and growth hormone (GH) were all normal. Glycosylated hemoglobin (HbA1c) was 5.60% (reference range: < 6.4%). Adrenal hormone, thyroid hormone, and gonad hormone were all normal. The oral glucose tolerance test (OGTT) and insulin and C-peptide release tests showed that the patient’s level of insulin and C-peptide secretion were high (**Table 1**). According to the above examination results, the initial diagnosis was hypoglycemia. However, whether the cause was insulinoma, insulin autoimmune syndrome, or insulin resistance needed further examination. Pancreatic MRI indicated a 6 mm diameter nodule-like protrusiona in pancreatic neck. (**Figure 2A**) and endoscopic ultrasonography (EUS) indicated a low echo area in the pancreatic neck (**Figure 2B**). According to **Figures 2A, B**, insulinoma seems to be the cause of the diagnosis. To further clarify the diagnosis, endoscopic ultrasonography-fine needle aspiration (EUS-FNA) and anaqueous alcohol ablation was performed in the gastroenterology department of our hospital. According to the postoperative disease examination (**Figure 2C**) and the review of OGTT and islet function (**Table 2**), insulinoma was ruled out as a diagnosis. To further clarify the cause, further genetic testing was needed. The patient and parents signed informed consent. The hospital ethics committee approved the study.

**Table 1:** Results of the parental OGTT for patient and parents.

Time (h)	BG (mmol/L)			Insulin (U/mL)			C-P (ng/ml)		
	Patient	Father	Mother	Patient	Father	Mother	Patient	Father	Mother
0	3.84	5.27	4.78	8.30	10.10	4.54	11.36	1.35	1.37
0.5	8.30	12.44	6.92	243.50	147.00	66.27	83.00	7.75	7.14
1	11.36	14.22	7.31	451.50	250.20	77.13	12.42	12.32	11.33
2	12.10	9.63	6.19	609.30	503.00	64.19	454.50	18.12	11.12
3	6.96	1.41	5.32	762.40	106.70	52.87	16.46	5.30	10.93
4	3.01	–	–	413.70	–	–	6.64	–	–
5	2.93	–	–	111.80	–	–	2.34	–	–

OGTT, oral glucose tolerance test; BG, blood glucose; C-P, c-peptide.

**Table 2:** Results of the OGTT review after performing EUS-FNA and after oral metformin.

Time (h)	BG (mmol/L)		Insulin (U/mL)		C-P(ng/ml)	
	After EUS	After metformin	After EUS	After metformin	After EUS	After metformin
0	6.23	4.64	>1000.00	3.70	12.25	2.89
0.5	8.61	7.15	>1000.00	20.90	154.0	5.89
1	11.38	9.14	>1000.00	18.10	16.32	7.03
2	13.66	7.00	>1000.00	14.50	19.80	3.65
3	9.93	4.56	>1000.00	10.00	21.12	2.24
4	8.64	5.02	>1000.00	8.00	15.67	3.80
5	3.88	4.50	897.90	7.40	9.62	5.65

OGTT, oral glucose tolerance test; BG, blood glucose; C-P, c-peptide; US, endoscopic ultrasonography; EUS-FNA, endoscopic ultrasonography-fine needle aspiration.

**Figure 3:**
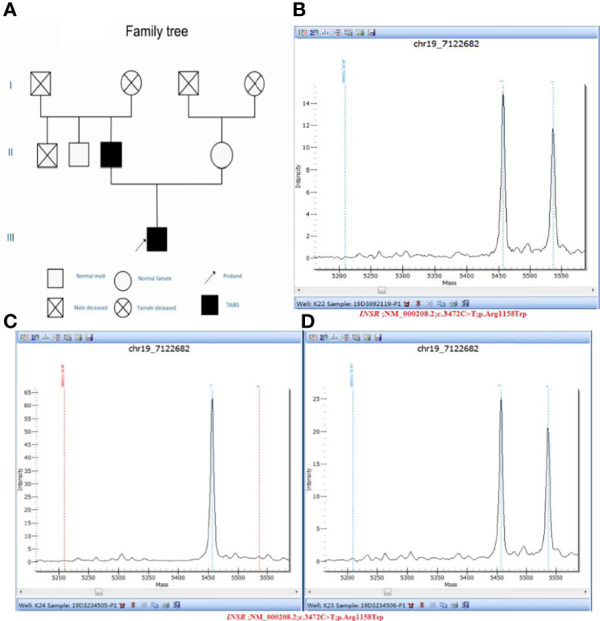
**(A)** Pedigree diagram and verification results of genetic testing for the proband and his parents: **(B)** Sample number: 19D3692119, Affiliation: proband, Nucleotide change: c.3472C>T, Amino acid changes: p.Arg1158Trp, Verification result: heterozygosis, Test method: Mass spectrometry validation; **(C)** Sample number: 19D3234505, Affiliation: mother, Nucleotide change: normal, Amino acid changes: normal, Verification result: normal, Test method: Mass spectrometry validation; **(D)** Sample number: 19D3234506, Affiliation: father, Nucleotide change: c.3472C>T, Amino acid changes: p.Arg1158Trp, Verification result: heterozygosis, Test method: Mass spectrometry validation.

Clinical whole exon gene sequencing (WES): 3 ml of whole blood was drawn from the patient and selected family members and was sent to the Clinical Laboratory Center of Shenzhen Huada for processing. Blood samples from other family members were not available because they were not local. The detailed steps were as follows:(1) a Covari-LE220 ultrasonic instrument (Massachusetts Company. USA) DNA was interrupted and library preparation was tested by Agilent2100 and BMG; (2) we captured DNA from the clinical whole exome; (3) using the high-throughput BGISEQ-500 platform, gene fragments of the identified mutated regions were subjected to upstream and downstream primer design and PCR amplification. The products were subjected to mass spectrometry or Sanger sequencing, and the results were aligned to the INSR gene standard series to validate the gene microarray results of capture and high-throughput sequencing. Finally, INSR was detected in the peripheral blood DNA of the patient and the father: c.3472C>T(p. Arg1158Trp) Variability (**Figure 3**). According to patient’s symptoms, signs, OGTT,islet function, and genetic test results, TAIRS was considered as the cause of the diagnosis, the proband’s parents’ OGTT, and islet function examination (**Table 1**) found that his father also had insulin resistance. After giving oral metformin (500mg, three times in a day, oral before meal) for two years, islet function (**Table 2**) suggested that the insulin levels, the incidence of nocturnal hypoglycemia, and preprandial hypoglycemia decreased significantly without additional meals.

## Discussion

TAIRS is an autosomal recessive or dominant genetic disease caused by the INSR gene mutation, which is a rare disease. More than 20 cases are reported abroad and only a few cases are reported in China. Since Yoshiransa et al. (1) and Kadowaki et al. in 1988 (2) reported mutations in the insulin receptor gene in patients with congenital insulin resistance syndrome, it has been recognized that any link of the insulin signaling pathway can cause insulin resistance. One of the most important links is the insulin receptor. The INSR gene is in 19P13.2-13.3 and is approximately 170 kb. Made up of 22 exons and 21 introns, it is a tetramer composed of 2 α subunits and 2 β subunits *via* disulfide bonds (3). Mutations in the INSR gene can cause a severe insulin resistance syndrome, clinically characterized by hyperinsulinemia, impaired insulin resistance and glucose tolerance, acanthosis nigricans, hyperandrogenemia, and high adiponectin levels (4). However, according to some symptoms, it is divided into Leprechauns syndrome (Donahue syndrome), Rabson-Mendenhall syndrome, and type A insulin resistance syndrome. TAIRS tends to be mild and patients can survive to adulthood, with some potentially succumbing to chronic complications of diabetes. However, some patients with TAIRS get better as the disease progresses (5). The INSR gene mutations identified have more than 90 different sites, including missense, nonsense mutation, insertion, deletion mutation, composite rearrangement, and more than 20 genes related to TAIRS mutation. Taylor suggested that INSR gene mutation can lead to INSR synthesis, transport barriers, reduce binding capacity, decrease tyrosine kinase activity, and accelerate enzyme degradation (6), thus causing a series of clinical symptoms. Among them, the main pathogenesis of TAIRS is β subunit mutation of the INSR gene, which mostly shows a decrease or loss of tyrosine kinase activity but can also be present in diabetic patients or in the normal population (7). Currently, there is no clear diagnostic standard for TAIRS in China and abroad. The diagnosis is based on whether the patient has hyperinsulinemia, acanthosis nigricans, and hyperandrogenemia. At the same time, attention should be paid to the differential diagnosis of hyperinsulinemia, excluding other diseases such as insulinoma, and insulin autoimmune syndrome. Insulinoma often presents with typical Whipple triad: (1) hypoglycemia, (2) blood glucose at onset is lower than 2.8mmol/L, (3) symptoms disappear immediately after oral or intravenous glucose injection. Insulin autoimmune syndrome is rare in clinical practice. The main feature is that exogenous insulin is not used but high concentration of immunoactive insulin and high-potency insulin autoimmune antibodies appear in the blood, causing spontaneous hypoglycemia. and at the same time.This patient at puberty, mainly due to repeated hypoglycemia, OGTT after admission found obvious hyperinsulinemia and negative insulin-related antibody does not consider insulin autoimmune syndrome. Pancreatic MRI and ultrasound gastroscopy revealed pancreatic space occupation, which was highly suspected as insulinoma. EUS-FNA and anhydroalcohol ablation was performed in the Department of Gastroenterology under endoscopic ultrasonography. Postoperative examination and reexamination of insulin level did not support insulinoma and the patient and his family agreed to detect the INSR gene. According to patient’s symptoms, signs, OGTT, islet function, and genetic test results, TAIRS was considered as the cause of the diagnosis. This study reviews a severe hyperinsulinemia pedigree with clinical TAIRS, and direct sequencing of the clinical whole exome of this patient revealed heterozygous missense mutation R1158 W (CGGTGG) in exon 19 of the INSR gene. This mutation was reported by Longo N et al. (8) in 1999 and Takasawa K et al. (9) in 2019, both confirming that this mutation leads to accelerated insulin receptor degradation and impaired activation of receptor autophosphorylation of insulin activation. The proband’s OGTT in this study showed low fasting glucose, slightly higher postprandial glucose, simultaneous insulin levels, and insulin-C peptide release tests showed endogenous hyperinsulinemia. Because the maintenance of fasting blood glucose levels mainly depends on the output of liver glucose and hyperinsulinemia suppresses this function of the liver, severe insulin resistance to peripheral glucose utilization caused by INSR mutation and postprandial hyperglycemia, excessive insulin secretion and slow insulin degradation caused by meal stimulation, led to the occurrence of paroxysmal premeal hypoglycemia. In addition, hyperinsulinemia caused by severe insulin resistance activates the IGF receptor, causing accelerated metabolic growth of skin keratinocytes or fibroblasts, epidermal cell proliferation, and clinical acanthosis nigricans (10). At the same time, hyperandrogenemia may occur in TAIRS, which can be associated with polycystic ovary syndrome (PCOS) but the patient had normal gonad hormones. Although he was hirsutic, there was no hairline shift or acne, so it was possible that hyperandrogenemia was more likely to occur in young women. To study the genetic pattern of the insulin receptor gene mutation in this proband and the relationship between the genotype and the clinical phenotype in this pedigree, this study simultaneously determined the exon DNA sequence of exon 19 of the parental insulin receptor. The results showed that the father also had a heterozygous R1158 W missense mutation, while the mother was normal. The father denied a history of diabetes and the OGTT and insulin release tests also suggested endogenous hyperinsulinemia. However, the insulin levels of the proband were three times higher than his father and the clinical manifestations of insulin resistance were more severe than his father, suggesting that environmental factors, except INSR gene mutations or other gene mutations, may cause insulin resistance. It was worth noting that only 10% of all the TAIRS patients had INSR gene mutations, as well as other genes, such as encoding nuclefibrillin A, which can also lead to TAIRS (11). Whether the proband’s father had hypoglycemia or diabetes also required long-term follow-up. It should be pointed out that clinical manifestations of insulin resistance, such as acanthosis nigricans, are related to the severity of insulin resistance and are not determined by other specific genetic mutations. Thus, the proband’s father did not present black echinothosis manifestations. In addition, in the course of clinical whole-exome testing of the proband, the pathogenic gene MUTYH causing familial adenomatous polyposis type 2(T2FAP), had heterozygous missense mutations: c.857G>A (p. Gly286Glu). The Sanger point mutation of the proband’s father are autosomal recessive, homozygous, or compound heterozygous pathogenic variants which can cause disease, mainly characterized by adult-onset multiple colorectal adenoma and adenomatous polyposis, with a significantly increased risk of colorectal cancer (12). Further studies are needed to determine whether TAIRS and T2FAP are genetically correlated.

TAIRS has no specific drugs for treatment. In general metformin is given orally to improve insulin resistance while preventing long-term complications of diabetes and reducing the effects of hyperandrogenemia (13). TAIRS has a relatively good prognosis and Taylor et al. (6) reported that the prognosis is associated with the location or number of mutations in the INSR gene loci. Therefore, gene diagnosis is crucial and the gene diagnosis and gene mutation sites should be actively defined; the insulin receptor gene mutation site is located at codon 1158 of the subunit. The type of mutation is a missense mutation. Two similar cases were reported in this site (8, 9), which is the first in China, proving that this site mutation causes reduced tyrosine kinase activity and decreased ability to bind insulin to insulin receptors, resulting in possible insulin resistance syndrome (14, 15). Takasawa K et al. (9) reported six patients with TAIRS and four had mutations in the INSR gene and two others without mutations, further suggesting that all patients with insulin resistance did not have INSR gene mutations. Huang Zhimin et al. (16) followed one patient for 7 years, which showed a downward trend in the overall insulin secretion levels over time. However, it was not accompanied by the simultaneous deterioration of blood glucose. Hatteraley et al. (17) showed that islet cell function will eventually fail over time and patients with TAIRS will face chronic complications of diabetes. At present, the use of oral metformin (500mg, three times in a day, oral before meal) for two years, significantly reduced the number of hypoglycemia episodes. However, the later progress of islet function still requires further follow-up of the patient’s condition to guide future clinical work.

## Conclusions

TAIRS is rare and clinically insidious with a relatively good prognosis. The diagnosis is mainly determined by symptoms, signs, islet function, and INSR genetic testing. Meanwhile, other diseases, such as hyperinsulinemia, insulinoma, and insulin autoimmune syndrome, should be actively excluded to avoid misdiagnosis or missed diagnosis. In treatment, oral metformin is the main treatment, which is also crucial to monitoring islet function and later outpatient follow-up. In clinical practice, the understanding of TAIRS should be strengthened to achieve early detection, early diagnosis, early treatment to improve patient prognosis, and increase the possibility of survival. These findings suggest that metformin is a useful therapeutic option for treating type A insulin resistance syndrome.

## Data Availability Statement

The datasets for this article are not publicly available due to concerns regarding participant/patient anonymity. Requests to access the datasets should be directed to the corresponding author.

## Ethics Statement

The studies involving human participants were reviewed and approved by Medical Ethics Committee of Yichang First People’s Hospital. The patients/participants provided their written informed consent to participate in this study. Written informed consent was obtained from the individual(s) for the publication of any potentially identifiable images or data included in this article.

## Author Contributions

All the authors have contributed significantly. JiY designed the study. WY and LW wrote the manuscript. WY, LW, YL, WenW, LZ, WeiW, JuY, and FC collected and analyzed the clinical data, participated in discussion. JiY supervised the study and corrected the manuscript. All authors contributed to the article and approved the submitted version.

## Funding

This research was supported by the Yichang Scientific Research and Development Project (No. A12-301-23 and No. A18-301-32).

## Conflict of Interest

The authors declare that the research was conducted in the absence of any commercial or financial relationships that could be construed as a potential conflict of interest.

## Publisher’s Note

All claims expressed in this article are solely those of the authors and do not necessarily represent those of their affiliated organizations, or those of the publisher, the editors and the reviewers. Any product that may be evaluated in this article, or claim that may be made by its manufacturer, is not guaranteed or endorsed by the publisher.
